# Evaluation of Standard-of-Care Practices Among Physicians Who Treat Other Physicians: A Qualitative Study

**DOI:** 10.1001/jamanetworkopen.2022.36914

**Published:** 2022-10-18

**Authors:** Anna McNair Avinger, Tekiah McClary, Margie Dixon, Rebecca D. Pentz

**Affiliations:** 1 Winship Cancer Institute, Emory University, Atlanta, Georgia; 2 Department of Hematology/Oncology, Emory University School of Medicine, Atlanta, Georgia

## Abstract

**Importance:**

Ethical discussions have suggested that physicians who treat other physicians may put their physician-patients at risk of receiving non–standard-of-care treatment, which may result in worse outcomes. This phenomenon occurs when a physician treats a fellow physician as a VIP (very important person), and is therefore known as *VIP syndrome*. It is important to assess physicians’ perceptions when treating physician-patients.

**Objective:**

To determine whether the physicians treating other physicians have attitudes toward or act in ways that could place physician-patients at risk for VIP syndrome.

**Design, Setting, and Participants:**

This 2-part qualitative study was conducted from December 1, 2021, to February 28, 2022. Physicians who worked at a single comprehensive cancer center with experience treating other physicians were eligible to participate. Convenience sampling was used. Emails and flyers were sent out with study information, and if interested, physicians were able to schedule an interview. Of 24 physicians responding, 3 did not have experience treating other physicians, yielding a sample of 21 (88%), which was sufficient to reach a saturation of themes. After the initial structured interview of physicians, follow-up key informant interviews were performed.

**Exposures:**

The structured interview was developed on the basis of a literature review and focused on factors that may contribute to VIP syndrome.

**Main Outcomes and Measures:**

Participant responses to open-ended questions were qualitatively coded using standard multilevel semantic analysis to assess physician perceptions of treating fellow physicians. A series of Likert-scaled questions were used to identify potential contributing factors to VIP syndrome.

**Results:**

Twenty-one physicians (11 men [52%], 11 White [52%], and 15 [71%] younger than 49 years) participated. Although no physician interviewed stated that they altered their usual treatment plans, 11 (52%) agreed that their physician-patients tried to dictate their own care, and 17 (81%) believed that their physician-patients obtained privileges, such as use of medical knowledge to participate in in-depth discussions of care, ability to obtain and use the treating physicians personal contact information, and receiving faster access to care. Eleven respondents (52%) reported increased stress, and 12 (57%) experienced more pressure not to disappoint their physician-patients.

**Conclusions and Relevance:**

The findings of this qualitative study suggest that when physicians treat other physicians, the physician-patients may obtain privileges unavailable to patients who are not physicians. Therefore, guidelines to help physicians navigate the complex relationships between themselves and their physician-patients are needed to ensure equitable outcomes between physician and nonphysician patients.

## Key Points

**Question:**

Do physicians deviate from standard-of-care practices when treating patients who are also physicians?

**Findings:**

This qualitative study of interviews with 21 physicians treating physician-patients found that 17 physicians (81%) agreed that their physician-patients were able to obtain privileges generally unavailable to nonphysician patients. None of the physicians interviewed stated that they altered their treatment plans for physician-patients.

**Meaning:**

These findings suggest that guidance and education are needed to help physicians navigate the complexities of treating other physicians.

## Teaser

This qualitative study explores potential factors that may contribute to deviation from standard of care when physicians treat patients who are also physicians.

## Introduction

Weintraub^1^ first defined patients who would be considered very important persons (VIPs) in 1964 as “any patient who has been able either through personal influence or professional status to exert unusual pressure upon [hospital] staff.” Other definitions followed. In 2007, Mariano and McLeod^2^ defined a VIP as “any patient that causes the physician to feel intimidated (ie, experience anxiety or tachycardia),” and in 2016, Alfandre et al^3^ defined VIPs as “very influential patients whose individual attributes and characteristics, coupled with their behavior, have the potential to significantly influence a clinician’s judgment or behavior.” These various definitions leave room for interpretation about who exactly is a VIP; they are often considered celebrities or politicians, but they can also be friends and colleagues. In this study, we attempted to gain a better understanding of the dynamics between physicians and their patients who are also physicians.

When a VIP receives non–standard-of-care (non-SOC) treatments, which may result in worse outcomes, the phenomenon is termed *VIP syndrome*.^2^ The presence of VIP syndrome in the treatment of physician-patients is not well known. In 1 study on childbirth,^4^ physician-mothers, who are more likely to be knowledgeable about treatment options, had better health outcomes than comparable non–physician-mothers. However, other studies^5,6^ have reported that special treatment of physician-patients, such as giving personal contact information, avoiding uncomfortable testing, or increasing pressure on the treating physician, can create challenges for physicians treating other physicians that may result in deviations from SOC. Weintraub^1^ stated that giving the patient privileges is the first element of the VIP syndrome and included the following privileges in his report: waiving routine admission procedures, regular communication with top administrators, special scheduling accommodations, private rooms, unique visiting hours, and different diets. In the present report, we define *privilege* as any difference in treatment given to physician-patients that deviates from SOC practices and procedures, with the intention of recognizing or because of the special position of the physician-patient. Additional differences in care between physician-patients and non–physician-patients described in the literature include differences in medical record documentation protocols, communication of results, scheduling,^7^ using medical jargon, and engaging the physician-patient more in treatment decision-making.^8^

Although the descriptions of VIP syndrome can be interpreted to imply that physician-patients are VIPs, empirical evidence that explores the presence of VIP syndrome in the treatment of physician-patients is needed.^1,9^ This study explored whether the factors that may trigger VIP syndrome are present, making treating physicians vulnerable to deviating from SOC treatment for their physician-patients.

## Methods

This qualitative study was approved by the Winship Cancer Institute Protocol Monitoring and Review Committee and by the Emory University Institutional Review Board. All participating physicians provided verbal informed consent. The Consolidated Criteria for Reporting Qualitative Research (COREQ) guidelines were followed.

We designed a qualitative descriptive study^10^ that consisted of 2 parts: (1) an initial structured interview of physicians practicing at a cancer center, and (2) follow-up with key informant interviews among previously interviewed physicians. Any physician working at Winship Cancer Institute in Atlanta, Georgia, between December 1, 2021, to February 28, 2022, who had experience treating 1 or more physician-patients was eligible for the study. Using convenience sampling, we emailed the 78 physicians who participate in the Disease Site Working Groups, using a list obtained from the cancer center administration. The Disease Site Working Group list was chosen because the interviewer attends these meetings, and the physicians would recognize the sender of the email. The mass email included information about the study, and if interested, physicians were able to schedule an interview via telephone, videoconference, or in-person, expected to take 20 to 30 minutes to complete. Before beginning the interview, the interviewer (A.M.A.), an ethics fellow doing a year-long research internship at the cancer center, explained the study in detail, shared the consent form, and obtained verbal consent. The consent included a query whether the participant would agree to be contacted for a follow-up key informant interview. Only those who agreed were contacted for the key informant interview. Answering questions in the interviews documented consent. We planned to enroll participants until saturation of themes was reached, which was usually accomplished with 15 to 20 participants.^11^

### Structured Interviews With Physicians

The questionnaire was designed based on a review of the literature on VIPs and VIP syndrome. It was cognitively tested with 2 medical students, 1 professor of bioethics, and 3 other ethics researchers to assess clarity and completeness before finalization.^12^ The interview started with open-ended and multiple-choice questions, which asked about the physician’s experience of treating physician-patients, specifically the treating physician’s feelings, time spent, and attention given and whether different treatments were offered. The interview proceeded to a set of 10 Likert-scaled questions designed to supplement our interview data about whether the potential contributing factors to VIP syndrome were experienced.

We developed the Likert scale interview questions based on the elements of VIP syndrome identified in the literature that may lead to not providing SOC tests and treatments and worse outcomes. The Likert scale questions were designed to provide further insight into the qualitative interviews and not as statistically significant results, owing to the small sample size needed for saturation of themes. The following elements of VIP syndrome were analyzed in the Likert-scale portion of the questionnaire:

“The patient is able, through the application of external pressures, to wrest from the hospital certain privileges.”^1^
“The hospital staff and patients withdraw from the VIP.”^1^
Treating physicians experience greater levels of anxiety when treating physician-patients, which may lead to differences in care and ultimately bad outcomes.^2,7,8,9^
“The VIPs themselves are part of the problem. Because of their position and status, they expect special care.”^2^
VIP patients try to demand or control their own care.^2,8^
“Deviations from the usual practice of medicine occur. The usual procedures are bypassed or abbreviated….”^2^
“An atmosphere of intimidation prevails. The physician strives to avoid disapproval and may actually be eager to court the approval of the VIP…. Procedures and tests that are uncomfortable may be postponed or never done, in the effort to avoid subjecting the VIP to inconvenience or pain.”^2^

Interviews were audiorecorded. The open-ended questions were qualitatively coded using standard multilevel semantic analysis, working directly from the audiorecording.^13^ After the first 5 interviews, major themes and types of answers were identified independently by 2 researchers (A.M.A. and T.M.). All discrepancies were resolved by consensus, and the code book was then finalized. All interviews were then coded using the code book (A.M.A.) and double coded (T.M.), with discrepancies resolved by consensus. Codes were entered into an Excel spreadsheet (Microsoft Corporation). Frequencies of major themes mentioned were calculated (A.M.A.) and double-checked by the principle investigator (R.D.P.).^14^

The frequency of response to the Likert scale questions were tallied with “strongly agree” and “somewhat agree” combined and “strongly disagree” and “somewhat disagree” combined. Neutral answers were tallied as well. Descriptive statistics were used to summarize the demographic characteristics and responses to the interviews.

### Key Informant Interviews With Physicians

The key informant open-ended questions were created after all initial interviews were conducted to gain clarity on the following issues:

- Do physician-patients deserve any kind of special treatment?
- What privileges are acceptable to give to physician-patients?
- What natural advantages do physician-patients have?
- What privileges or advantages would physicians expect to have if they became a patient at the institution where they work?

Key informant interviews were conducted with a subset of the physicians who completed the initial interview and had consented to be contacted. The interviews were analyzed using the same methods as the initial interviews.

## Results

### Initial Interviews

Twenty-four physicians responded to the mass email. Three physicians approached did not have experience treating other physicians and were therefore ineligible to participate, resulting in 21 interviews (88%) completed (11 men [52%] and 10 women [48%]; 15 [71%] younger than 49 years). In terms of race and ethnicity, 7 (33%) were Asian, 2 (9%) were Middle Eastern, and 11 (52%) were White. All demographic data were collected by self-report. Race and ethnicity could not be analyzed in such a small sample. The interviews were conducted from December 1, 2021, to February 28, 2022. No participant terminated the interview early. All 21 of the participating physicians had experience treating more than 1 physician-patient. The demographic characteristics of the participating physicians are described in Table 1.

**Table 1.** Demographic Characteristics of Participating Physicians

Characteristic	No. (%) of participants (n = 21)^a^
Sex	
Women	10 (48)
Men	11 (52)
Age, y	
30-39	6 (29)
40-49	9 (43)
50-59	1 (5)
60-69	2 (9)
70-79	2 (9)
Unknown	1 (5)
Specialty	
Hematologic oncology	4 (19)
Medical oncology	13 (62)
Surgical oncology	2 (9)
Neurosurgery	1 (5)
Urology	1 (5)

^a^ Percentages have been rounded and may not total 100.

Responding to the open-ended questions, 13 of 21 physicians (62%) expressed negative feelings when treating other physicians, such as stress, awkwardness, and feeling judged. Nine physicians (43%) expressed positive feelings when treating other physicians, such as feeling honored, comfortable, and more empathetic. Thirteen physicians (62%) described some difference in their usual patient care when treating physician-patients as opposed to non–physician-patients. First, 10 physicians (48%) said they did not consult with more colleagues for physician-patients; however, 7 (33%) said they consulted with more colleagues if the physician-patient asked them to. Second, 10 physicians (48%) said they gave physician-patients their personal contact information, or the physician-patients already had it. Third, 18 physicians (86%) explained that their conversations with physician-patients were different than those with non–physician-patients. Fourteen of these physicians used more advanced medical language, and 9 described speaking in more depth about research and data with their physician-patients. No physicians reported offering different treatments or tests to their physician-patients compared with other patients.

The Likert scale responses provided further support that these physicians experienced many of the factors that can contribute to VIP syndrome (Table 2).^1,2,3,5,7,8,9,15,16,17,18^ For example, 11 physicians (52%) agreed that they experienced increased stress when treating physician-patients and pressure not to disappoint their physician-patients. Interestingly, 17 physicians (81%) reported that physician-patients were able to obtain certain privileges that other patients would not be given.

**Table 2.** Physician Responses to Likert Scale Statements

Likert scale statements	Physician responses, No. (%) (n = 21)		
	Agree	Neutral	Disagree
My physician-patient(s) were able to obtain certain privileges that would not be given to other patients^1,2^	17 (81)	1 (5)	3 (14)
I felt withdrawn from my physician-patient(s)^1^	0	2 (10)	19 (90)
I felt that other hospital staff were withdrawn from my physician-patient(s)^1^	5 (24)	2 (10)	14 (67)
I felt increased stress when treating my physician-patient(s)^2,7,15,16^	11 (52)	2 (10)	8 (38)
I felt pressured to provide special treatment to my physician-patient(s)^7,9,15,16,17^	7 (33)	4 (19)	10 (48)
I felt that my physician-patient(s) tried to demand or dictate their own care^2,9,16^	11 (52)	1 (5)	9 (43)
I felt pressure from hospital administration or colleagues to meet the physician-patient’s demands^9,17^	8 (38)	1 (5)	12 (57)
My usual approach to care differed in some way when treating physician-patient(s)^5,7,8,18^	5 (24)	2 (10)	14 (67)
I felt more pressure not to disappoint my physician-patient(s) than I did with other patients^2^	12 (57)	1 (5)	8 (38)
My patient’s role or previous role as a physician influenced his/her/their medical care overall^3^	12 (57)	2 (10)	7 (33)

Three privileges and/or advantages were most frequently identified by physicians during the qualitative interviews. First, having medical knowledge, which we define as the ability to understand medical terminology; awareness of medical procedures, symptoms, and treatment; and professional experience in the medical field. Medical knowledge leads to more in-depth discussions and sharing of technical educational tools that may result in increased understanding and better-informed decision-making. Second, physician-patients were able to obtain and use the treating physician’s personal contact information. Third, faster scheduling and quicker access to care were available to physician-patients. These and additional privileges are listed in the Figure.

**Figure.**
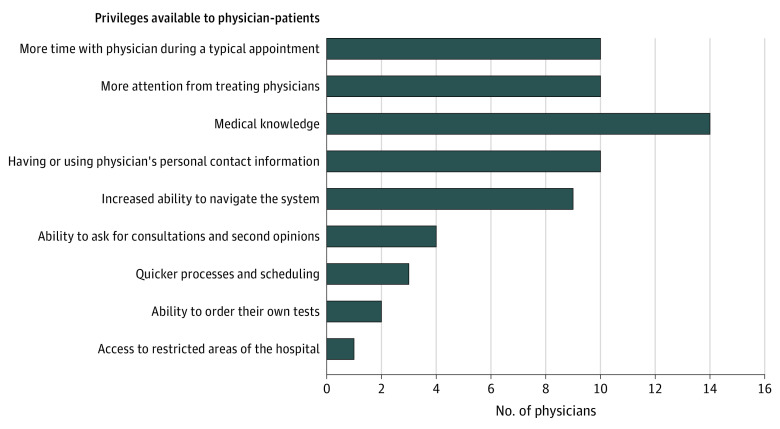
Physicians Who Identified Privileges Obtained by Their Physician-Patients A total of 21 physicians were included in the results.

### Key Informant Interviews

Physicians were split into the following 3 groups, based on responses to the 10 Likert scale questions about contributing factors to the VIP syndrome:

- Low risk of VIP syndrome: These physicians agreed to experiencing 0 to 3 of the contributing factors.
- Moderate risk of VIP syndrome: These physicians agreed to experiencing 4 to 6 of the contributing factors.
- High risk of VIP syndrome: These physicians agreed to experiencing 7 to 10 of the contributing factors.

Seven physicians were chosen for follow-up interviews: 2 physicians from the group at low risk, 3 from the group at moderate risk, and 2 from the group at high risk. The interviews were conducted 1 month after the initial interviews were completed. When we asked what advantages the physician-patients had, the 2 main advantages were physician-patients obtained and used their treating physician’s personal contact information (6 of 7 physicians mentioned this), and physician-patients had greater access to care (eg, scheduling priority, system workarounds; 5 of 7 physicians mentioned this). This second advantage likely encompasses quicker scheduling, faster access to care, and ability to ask for consultations and second opinions, as described in the Figure.

### Privileges

Physicians demonstrated differing opinions about whether physician-patients should have certain privileges or advantages. In the initial interview, of the 17 physicians who agreed that physician-patients are able to obtain certain privileges, 5 (29%) mentioned concerns about the health care system, and how it is more difficult for non–physician-patients to advocate for themselves, navigate the complex call centers, take advantage of resources, and so forth. In contrast, 6 of the 17 physicians (35%) overall had no concerns because (1) giving physicians privileges is just “taking care of our own;” (2) the treating physicians could address issues if they arose; (3) the care and treatment are ultimately the same; and (4) the privileges are just natural advantages that come from the experience and knowledge one gains from working in health care. In addition, 2 of 17 physicians (12%) mentioned concerns about physician-patients abusing their personal contact information. Only 1 physician (6%) discussed concerns that physician-patients abuse their professional privileges as patients, such as arranging their own treatment.

In the key informant interviews, 5 of 7 physicians (71%) said that they think physician-patients deserve some level of professional courtesy. One physician (14%) said that physicians do not deserve special treatment, but that they get privileges naturally because of their experience and education in health care. This physician admitted to giving his personal contact information out and described using other physicians’ contact information to gain scheduling priority. Only 1 physician of 21 (5%) stood firm on his stance that every patient should be treated the same, with the same communication routes, similar education and terminology, scheduling, and so forth. Most physicians expressed some sort of conflict between wanting to treat every patient equally and fairly, but also recognizing that privileges do exist for physicians, and those privileges may not necessarily be wrong. Six of 7 key informant interviewees (86%) said they would use or have used privileges themselves, such as emailing or calling a physician to schedule an appointment for themselves or a loved one instead of going through the call center.

## Discussion

### VIP Syndrome

We found that there is significant complexity to the relationships between physicians and physician-patients. As shown in the Figure, participants reported that physician-patients are able to obtain special privileges, which is the first element of the VIP syndrome described by Weintraub.^1^ This raises the concern that physician-patients will be offered care that deviates from SOC and have worse outcomes. Interestingly, in our interviews, no physician described any differences in medical treatments or tests. This may indicate that SOC treatments were followed even when communication and educational procedures were altered. On the other hand, it may indicate that physicians are unaware that they are offering non-SOC tests or treatments to their physician-patients. Unfortunately, we were unable to determine whether the physician-patients experienced worse or better health outcomes due to their privileges. However, although we did not have access to patient outcomes, given our interview protocol, we were able to assess whether physicians treating other physicians exhibited risk factors for VIP syndrome. This is the first study, to our knowledge, that has empirically demonstrated that these risk factors exist for physicians treating physician-patients.

Our interviews with physicians also showed a lack of consensus on whether physician-patients should ethically be able to obtain privileges. Contradictions in the physicians’ answers when asked about the ethics of giving physician-patients privileges indicate the need for ethical discussion and guidelines for physicians treating other physicians.

Historically, professional courtesy has been defined as providing financial benefits to physicians or family members of physicians, such as waiving fees, offering discounts, and only billing the insurer.^19^ Based on a survey conducted by Levy et al^20^ in 1993, 96% of the 2224 physicians surveyed offered financial professional courtesy, although the 1965 Anti-kickback Statute prohibited any exchange of discounted care.^21^ Seventy-nine percent of these physicians who offered financial professional courtesy believed that it solidified bonds between physicians. Currently, this practice of professional courtesy is not as widely accepted or ethically supported.^22^ Although there are clear arguments against financial professional courtesy, whether or not the privileges our interviewees described are ethically appropriate has not been adequately analyzed. Multiple articles^6,8,18,19,21,22,23,24^ list the “dos and don’ts” of treating another physician that could be helpful to the practicing physician. Common suggestions from the literature are listed below:

1. All conversations about care and treatment should be documented in the medical record.^18^ Curbside conversations and treatment should be avoided.^23^
2. Physicians should acknowledge their emotions and assess whether they can provide objective and professional care. If emotional stress is too great, physicians should transfer the patient to another colleague who can provide more objective care.^8,18,19,24^
3. At all times, protect the confidentiality of the physician-patient.^21^
4. Treating physicians should acknowledge that the physician-patient is a physician and/or colleague, but they should not assume anything about what the patient already knows or does.^22^
5. Be aware that the physician-patient may be tempted to self-treat or skip follow-ups and advise the physician-patient not to do so.^6^

### Next Steps

Given that we did not assess outcomes, more studies need to assess and compare health outcomes for physician-patients with those of non–physician-patients, to determine empirically whether SOC is deviated from or whether the privileges given lead to better or worse outcomes. Thus, a future study could compare actual treatments and tests offered to and performed for physician-patients with those offered to and performed for a matched control group of non–physician-patients. Outcomes for both the physician-patients and the control group could then be compared. Because it may be possible that the physicians interviewed in this study were unaware they were in fact offering tests and treatments that are non-SOC, it would also be informative to compare the data on tests and treatments offered to physicians’ perceptions of how they treat physician-patients to test physicians’ self-awareness. This type of study would do much to advance our understanding of the presence of VIP syndrome in physician-patient care and could inform the creation of educational modules to address problems identified, including the treating physician’s blindness to his or her own deviations from prescribing SOC tests and treatments.

### Limitations

The major limitation of our study was not having access to assess patient outcomes, so we could not assess whether the worse outcomes associated with VIP syndrome affected the physician-patients. In addition, the study was performed at a single institution and only with oncologists, so the results may not be generalizable.

## Conclusions

The findings of this qualitative study suggest that physicians who treat other physicians may be at risk of VIP syndrome, because more than half of physicians agreed to the following statements: physician-patients are able to obtain certain privileges; physicians experience stress when treating physician-patients; physician-patients demand or dictate their own care; physicians experience pressure not to disappoint their physician-patients; and a physician-patient’s role as a physician influences his or her medical care overall. Guidelines and educational initiatives are needed to help physicians navigate the complex relationship between themselves and their physician-patients.
